# Content and Face Validation of Educational Infographics for Electronic Cigarette Cessation Among Malaysian Youth

**DOI:** 10.21315/mjms-04-2025-243

**Published:** 2025-10-31

**Authors:** Picholas Kian Ann Phoa, Jonathan Wiguna Halim, Yin How Wong, Charles Sharma Naidu, John Ing Kieng Hii, Faisal Athar Mohd Fadzil, Nurlaili Mohd Azizi, Lei Hum Wee

**Affiliations:** 1 School of Medicine, Faculty of Health and Medical Sciences, Taylor’s University, Subang Jaya, Selangor, Malaysia; 2 The Design School, Faculty of Innovation and Technology, Taylor’s University, Subang Jaya, Selangor, Malaysia; 3 Digital Health and Medical Advancement Impact Lab, Taylor’s University, Subang Jaya, Selangor, Malaysia; 4 Department of Creative Design, School of Architecture and Design, Faculty of Arts and Social Sciences, Sunway University, Subang Jaya, Selangor, Malaysia; 5 VORTEX XR Lab, Center for Future Learning, Taylor’s University, Subang Jaya, Selangor, Malaysia; 6 Centre for Community Health Studies (ReaCh), Faculty of Health Sciences, Universiti Kebangsaan Malaysia, Kuala Lumpur, Malaysia

**Keywords:** behavioural intervention, electronic cigarette, electronic nicotine delivery system, health education, preventive medicine

## Abstract

The increasing prevalence of electronic cigarette (e-cigarette) use among Malaysian youths highlights the inadequacy of current health education initiatives in addressing cessation challenges. This study aims to evaluate the content and face validity of educational infographics to support e-cigarette cessation among youth e-cigarette users in Malaysia. This study employed a cross-sectional study design and involved experts and youth e-cigarette users. Upon the development, 17 experts specialising in smoking or e-cigarette cessation, with a mean age of 39.10 years and a mean years of experience of 12.24 years, were recruited for content validation. Meanwhile, for the face validation, 10 Malaysian youth e-cigarette users, with a mean age of 24.00, were purposively selected to assess the clarity of the infographics and provide feedback. Content validity indices (CVI) and face validity indices (FVI) were calculated for each topic, with thresholds of > 0.74 and 0.83 indicating acceptable content. The infographics demonstrated strong content and face validity, with average CVI of 0.98 (range: 0.94, 1.00; 95% CI: 0.95, 1.00) and average FVI of 0.96 (range: 0.70, 1.00; 95% CI: 0.87, 1.00), respectively. The infographics can be utilised to promote e-cigarette cessation after the trial and implementation phase, to assess their real-world effectiveness.

## Introduction

Electronic cigarettes (e-cigarettes), with their diverse customisability and sleek designs, have become increasingly available commercially. This has caused a steady increase in the prevalence of e-cigarette use among Malaysians. In 2020, 5.4% of Malaysian adults aged 18 and above reported using e-cigarettes daily compared with 4.9% in 2019 and 0.8% in 2011 (1). Studies have also found that e-cigarette users were likely to be younger adults, males, students, have a higher education level, and live in urban areas (2, 3).

Nonetheless, e-cigarette cessation interventions remain inadequate and largely ineffective, leaving a critical gap in addressing this growing public health concern. Despite the alarming rise in e-cigarette use among younger populations, traditional stop smoking clinics still predominantly cater to older adults and fail to resonate with the unique preferences and behaviours of youths who are often plagued by high dropout rates and inconsistent engagement (4, 5). Therefore, e-cigarette cessation interventions should adequately address the psychosocial factors unique to e-cigarette use, such as user demographic, their level of knowledge and perception of e-cigarettes, substance use, mental health status, interpersonal social influences, media exposure, and laws and regulations (6). This lack of e-cigarette-specific, youth-centric cessation interventions underscores the urgent need for the development of specific approaches for e-cigarette cessation. Without addressing these gaps, current strategies risk perpetuating a cycle of ineffective interventions, leaving a vulnerable population at continued risk of nicotine addiction and its associated health consequences.

To address this issue, this study aims to develop infographics as a targeted intervention material on e-cigarette use, fostering greater motivation and success in e-cigarette cessation, and conduct content and face validity analyses involving both the experts in relevant fields and youth e-cigarette users to assess the relevance and clarity of the infographic’s content.

## Methods

### Study Design

A cross-sectional content validation study was conducted involving experts in behavioural intervention for smoking or e-cigarette cessation to evaluate the relevance of the infographics. Revisions were made upon review. Subsequently, a face validation study was conducted to assess the content clarity and comprehension among end users, specifically Malaysian youth e-cigarette users aged 18 to 25.

### Study Setting and Participants

Both the content and face validation studies were conducted online. For content validity, experts in behavioural interventions, smoking, e-cigarette cessation, and other relevant fields were recruited. The inclusion criteria comprised individuals with a minimum of one year of relevant background in research, academics, clinical practice, or other involvement in behavioural interventions, and with smoking or e-cigarette cessation. The participants should also be Malaysians and able to communicate in English. We excluded individuals who lack experience working with clients aged 18 to 25 years.

As for the face validity study, participants included current e-cigarette or dual (e-cigarette/conventional cigarette) users aged 18 to 25 years who were able to communicate in English. Individuals undergoing treatment or intervention for e-cigarette cessation, those who smoked only conventional cigarettes, or former e-cigarette users were excluded.

### Sampling Method and Recruitment Strategy

We targeted a minimum of nine experts to meet the acceptable CVI threshold of 0.78 for the content validity, as recommended by Lynn (7, 8). As for face validity, we targeted at least ten youths to meet the FVI threshold of 0.83, based on Mohamad Marzuki et al. (9, 10). Participants were recruited via purposive sampling, and the researchers identified relevant experts for content validity via online resources and networking. Prospective participants were contacted via email to explain the study procedures. In regard to e-cigarette smokers, recruitment posters were posted on social media and communication apps, and interested individuals contacted the researcher via email to participate in this study.

### Research Instruments and Data Collection

A content validity assessment form was developed to obtain experts’ evaluation on the relevance of the infographics using a four-point Likert scale (1 = Not relevant; 2 = Somewhat relevant; 3 = Quite relevant; and 4 = Highly relevant). Likewise, a face validity assessment form with a four-point Likert scale (1 = Not clear and understandable; 2 = Somewhat clear and understandable; 3 = Quite clear and understandable; and 4 = Very clear and understandable) was provided to e-cigarette users to assess the clarity and comprehensibility of the infographics. In addition, both experts and e-cigarette users were invited to provide written feedback for further refinement.

### Statistical Analysis

The data were recorded and coded in Microsoft Excel. The I-CVIs and I-FVIs were calculated using the following formula:


$$I-CVI=\frac{Number\;of\;experts\;rating\;the\;item\;as\;relevant}{Total\;number\;of\;experts},$$

or


$$I-FVI=\frac{Number\;of\;users\;rating\;the\;item\;as\;clear}{Total\;number\;of\;users}$$

Additionally, for the content validity study, the probably of chance agreement (pc) and the modified kappa (K) were calculated using the formulas below,


$$P_{c}=\frac{N!}{A!(N-A)!}\times 0.5^{N},$$

where,

*N* = total number of experts
*A* = number of experts rating the item as relevant
! = Factorial
0.5*^N^* = probability of randomly agreeing on an item (binary rating of “relevant” or “not relevant”)

and,


$$K=\frac{I-CVI-P_{c}}{1-P_{c}}$$

where,

I-CVI = item-content validation index
*P_c_* = probability of chance agreement

Open-ended responses were addressed individually and used pragmatically for revisions, and no qualitative analysis was performed. Subsequently, content with low CVI and/or FVI was revised in accordance with written feedback to produce the validated educational infographics.

## Content Validity

For the content validation, 17 out of 20 experts (85.0%) completed the survey. In terms of gender, the experts comprised 58.8% males (*n* = 10) and 41.2% females (*n* = 7), with ages ranging from 28 to 57 years and a mean age of 39.1 years. These experts were working as nurses, medical officers, medical specialists, psychologists, academicians in the fields of family medicine, public health, safety and health, and environmental health officers, with a mean experience of 12.24 years.

For the content validity analysis (Table 1), five of eight infographics obtained universal agreement (UA), with an I-CVI value of 1.00, and three infographics received an I-CVI value of 0.94. In general, all infographics were considered relevant with acceptable I-CVI values above 0.83, and the scale-level CVI based on the average method (S-CVI/Ave) is 0.98, with a 95% confidence interval of 0.95 to 1.00. Meanwhile, the scale-level content validity index based on universal agreement (S-CVI/UA) was 0.63, lower than the commonly accepted threshold of ≥ 0.80. In this regard, this index is known to be highly conservative, as a single expert’s disagreement can render an item invalid. Therefore, consistent with recommendations in the literature, our study emphasised the use of the S-CVI/Ave, which provides a more stable and representative estimate of content validity, particularly when a large panel of experts is involved (11). The *P_c_* values were very low, indicating minimal chance agreement, while the kappa values ranged from 0.94 to 1.00, reflecting excellent expert agreement beyond chance. Even though some of the experts did not provide additional comments in the open-ended section (Supplementary 1a), the feedback obtained was nonetheless used to revise the infographics.

## Face Validity

Upon revising the infographics following content validation, a face validation study was conducted, and feedback was received from 10 of 17 youth e-cigarette users sampled (58.8%). The e-cigarette users who completed and submitted the survey form consisted of 80.0% males (*n* = 8) and 20.0% females (*n* = 2), with ages ranging between 18 and 25 (M = 24.0 years).

**Figure 1:**
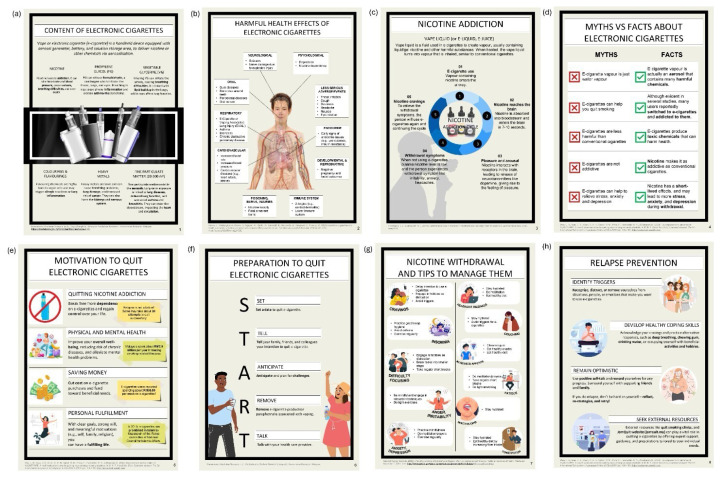
(a) Content of electronic cigarettes; (b) Harmful health effects of electronic cigarettes; (c) Nicotine addiction; (d) Myths vs. facts about electronic cigarettes; (e) Motivation to quit electronic cigarettes; (f) Preparation to quit electronic cigarettes; (g) Nicotine withdrawal and tips to manage them; and (h) Relapse prevention

For the face validity analysis (Table 2), the scale-level FVI based on the average method (S-FVI/Ave) was 0.96 with a 95% CI of 0.87 to 1.00, and the scale-level FVI based on the universal agreement method (S-FVI/UA) was 0.88. All but one infographic obtained UA, with an I-FVI value of 1.00. Infographic 1 “Content of Electronic Cigarettes” received an I-FVI value of 0.70. In the open-ended feedback section, two raters commented that the “fonts were too small,” and two raters suggested that the “words are very scientific” and “can be simplified” (Supplementary 1b). In response, the researchers increased the font size and simplified complex terms to enhance readability and comprehension. For example, “cytotoxic” was replaced with “toxic to organ cells,” “respiratory problems” with “breathing problems,” and “chronic” with “long-term.” These changes were made while ensuring the infographics remained accurate in conveying the intended information, resulting in the finalised infographics (Figure 1). The finalised infographics are presented in this article’s supplementary page (Supplementary 2: Guide to quit electronic cigarettes).

## Conclusion

In conclusion, the study has developed validated infographics grounded on a comprehensive literature review and current evidence. Although the materials are presented in a two-dimensional format, they can be adapted into various other forms, including videos, infographics, and three-dimensional media to enhance reach and engagement. Researchers and practitioners are encouraged to explore these adaptations in future studies and implementation research to assess their feasibility, acceptability, and effectiveness in real-world settings. In this regard, there is a substantial potential for innovation and broader impact that should be further explored.

## Supplementary Material

### Supplementary 1a and 1b: Open-ended Comments

**Table S1a:** Comments on the intervention material relevance by experts (*N* = 17)

Intervention materials	Comments and feedback
1: Content of electronic cigarettes	Please recheck if particulate matters are in e-cigarettes or in aerosols – E11 Vape or electronic cigarette is a smoking device with used with smoking substance with or without nicotine. Please refer to 852 Act – E17
2: Health effects of electronic cigarettes	“Adverse events” is usually associated with medications and does not tally with the title: “health effects.” Consider “harmful (health) effects?” Environmental effects can correlate to health, but not so clear here. Recent evidence also suggests risk of heart failure, cancer – E11 Words are quite jargon, for example, “APGAR score.” Also, in source reference, capitalise the journal name – E14
3: Nicotine addiction	Compared to the first material stating vape has no nicotine, a bit confusing here then. By “similar to conventional cig” means vapour = smoke? Different, right? Suggestion as follows: Vapour containing nicotine enters the airway and then bloodstream Blood carries nicotine to the brain Nicotine interacts with receptors in the brain leading to release of neurotransmitters, including dopamine, which gives rise to a feeling of pleasure When the plasma level of nicotine is low, withdrawal symptoms set in – E11For point number 2, please state how many seconds does it takes for nicotine to reach the brain – E14
4: Myths and facts about electronic cigarettes	Arrange facts vs myths as per title. For smoking cessation (SC), can say there’s evidence supporting SC using e-cig, but the majority maintained e-cig use after switching – E11 Nicotine releases GABA and B-endorphin, which reduce stress and anxiety (short-lived effects) – so not sure why it’s a myth – E11
6: Preparation to quit electronic cigarettes	Suggest: Remove e-cigarette related products or paraphernalia associated with vaping – E11
7: Nicotine withdrawal and tips to manage them	Not sure can state nicotine replacement therapy (NRT) for cravings when it is not yet approved in Malaysia – E11 For constipation, state increase fibre intake specifically, rather than healthy diet – E11
8: Relapse prevention	Please change Quitline to jomquit.moh.gov.my – E17

**Table S1b:** Comments on the intervention material clarity and comprehension by Malaysian university students who use e-cigarettes (*N* = 10)

Intervention materials	Comments and feedback
1: Content of electronic cigarettes	Fonts too small and make people lazy to read – R6 Understandable and very clear, but can simplified the word and focus to the point – R9 Font too small, words are very scientific, hard to understand – R10
2: Health effects of electronic cigarettes	Some are difficult to understand, especially endocrine and cardiovascular – R10
5: Motivation to quit electronic cigarettes	Some fonts are small and hard to read – R10

### Supplementary 2: Guide to Quit Electronic Cigarettes

Figure 2

Figure 3

Figure 4

## Figures and Tables

**Table 1:** Content validity indices of the infographics assessed by the panel of experts (*N* = 17).

Intervention materials	Experts in agreement (*N* = 17)	I-CVI	UA	*P_c_* (×10^−4^)	K
1: Content of electronic cigarettes	17/17	1.00	1	0.08	1.00
2: Health effects of electronic cigarettes	17/17	1.00	1	0.08	1.00
3: Nicotine addiction	16/17	0.94	0	1.40	0.94
4: Myths and facts about electronic cigarettes	17/17	1.00	1	0.08	1.00
5: Motivation to quit electronic cigarettes	17/17	1.00	1	0.08	1.00
6: Preparation to quit electronic cigarettes	16/17	0.94	0	1.40	0.94
7: Nicotine withdrawal and tips to manage them	16/17	0.94	0	1.40	0.94
8: Relapse prevention	17/17	1.00	1	0.08	1.00

S-CVI/Ave (95% CI)		0.98 (0.95, 1.00)			
S-CVI/UA			0.63		

I-CVI = item-content validation index; UA = universal agreement; *P*_c_ = probability of chance agreement; K = modified kappa; S-CVI/Ave = scale-content validation index/average; CI = confidence interval; S-CVI/UA = scale-content validation index/universal agreement

**Table 2:** Face validity indices of the infographics assessed by Malaysian university students who use e-cigarettes (*N* = 10)

Intervention materials	User in agreement (*N* = 10)	I-FVI	UA
1: Content of electronic cigarettes	7/10	0.70	0
2: Health effects of electronic cigarettes	10/10	1.00	1
3: Nicotine addiction	10/10	1.00	1
4: Myths and facts about electronic cigarettes	10/10	1.00	1
5: Motivation to quit electronic cigarettes	10/10	1.00	1
6: Preparation to quit electronic cigarettes	10/10	1.00	1
7: Nicotine withdrawal and tips to manage them	10/10	1.00	1
8: Relapse prevention	10/10	1.00	1

S-FVI/Ave (95% CI)		0.96 (0.87, 1.00)	
S-FVI/UA			0.88

I-FVI = item-face validation index; UA = universal agreement; S-FVI/Ave = scale-face validation index/average; CI = confidence interval; S-FVI/UA = scale-face validation index/universal agreement
