# Supplementary figures and images for: Content and Face Validation of Educational Infographics for Electronic Cigarette Cessation Among Malaysian Youth
Source: Malays J Med Sci. 2025 Oct 31;32(5):180–90. doi: 10.21315/mjms-04-2025-243 (PMC13132110; doi:10.21315/mjms-04-2025-243)

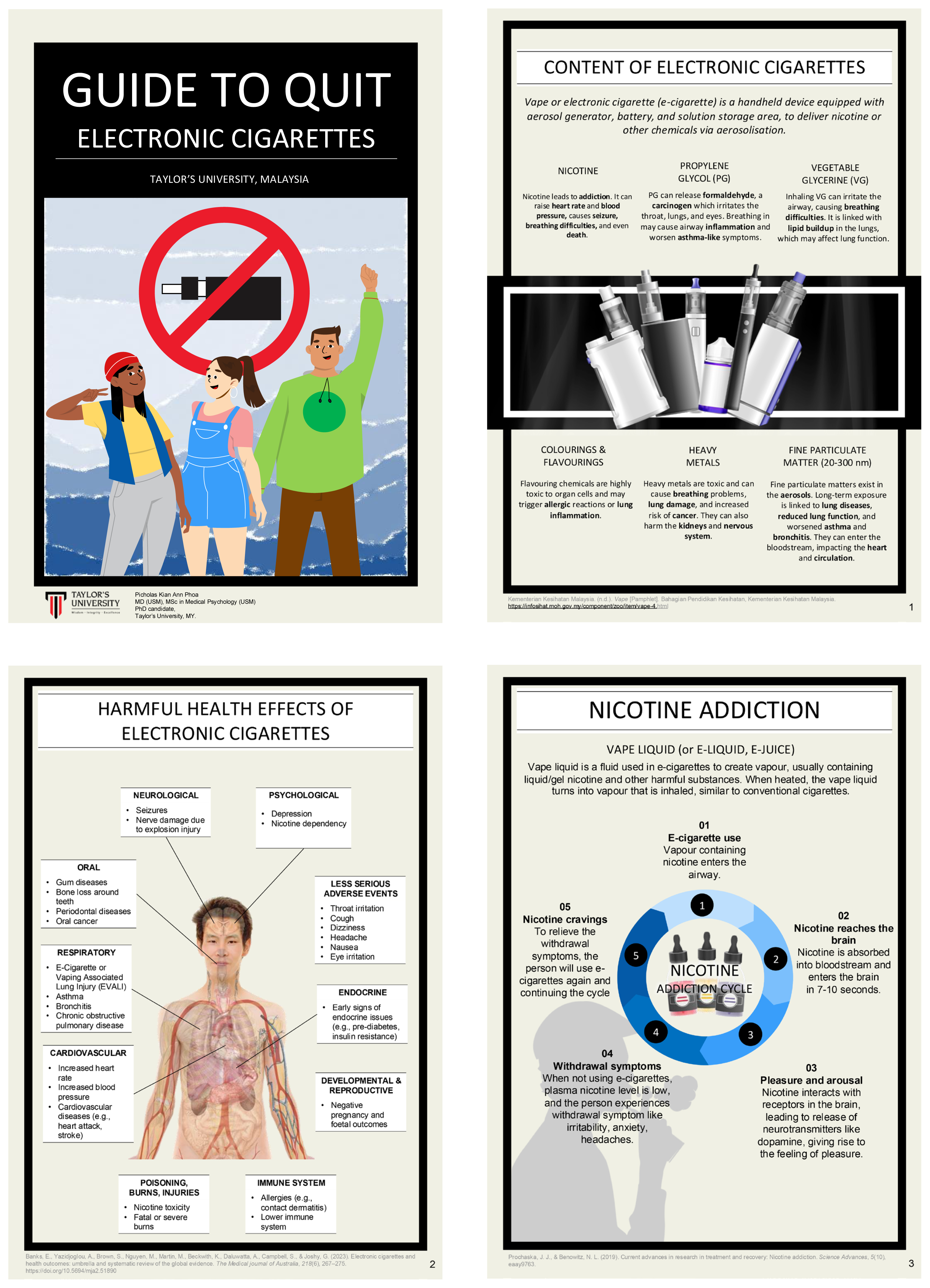

Supplement: Figure 2 [file 14mjms3205_bcs1.tif]

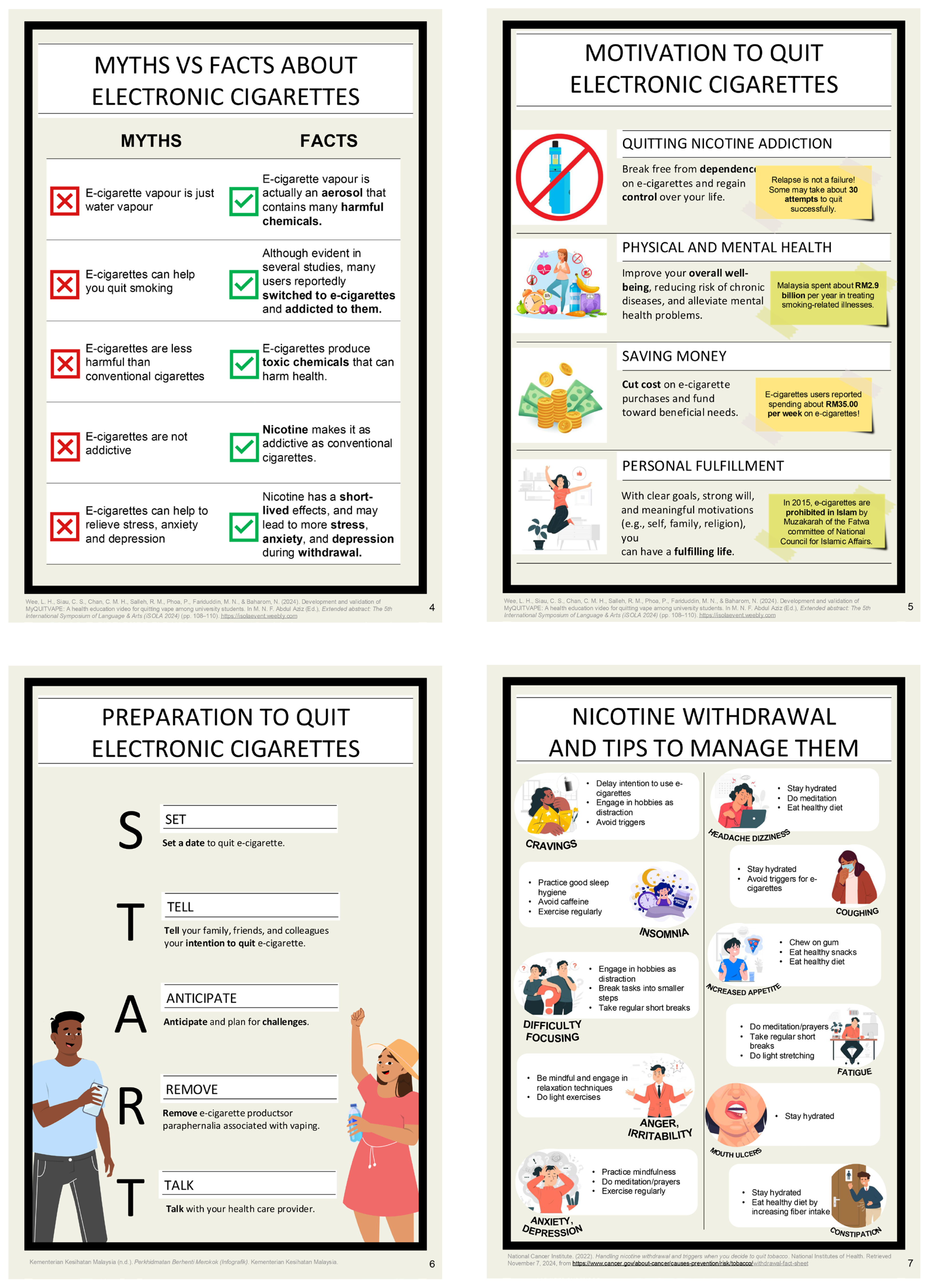

Supplement: Figure 3 [file 14mjms3205_bcs2.tif]

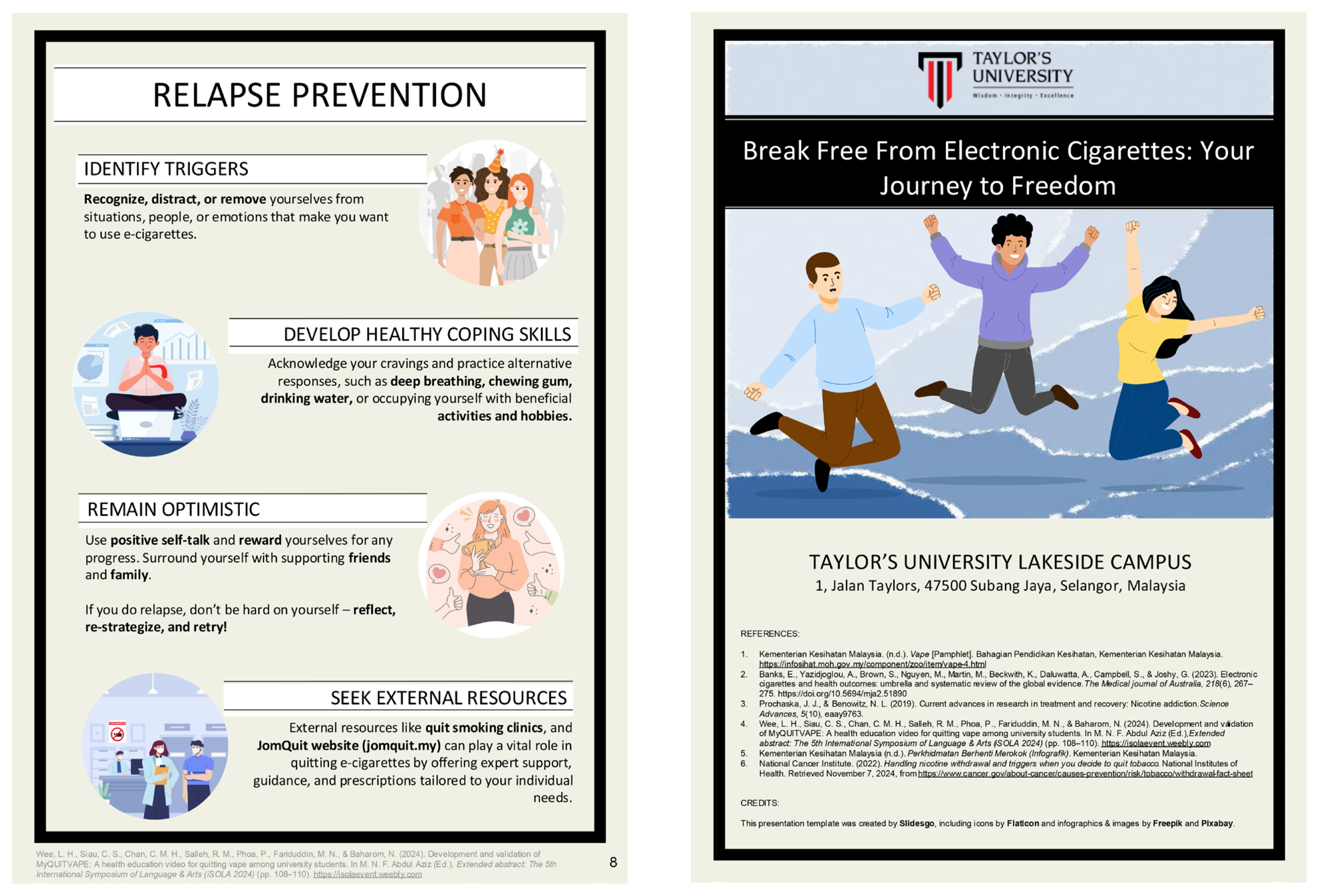

Supplement: Figure 4 [file 14mjms3205_bcs3.tif]
